# Autoreactive and parasite-specific IgG repertoires in asymptomatic and mild *Plasmodium falciparum* infection in Côte d’Ivoire

**DOI:** 10.1186/s13071-026-07508-9

**Published:** 2026-07-17

**Authors:** David Koffi, Nicolas Tchitchek, Fabien Herbert, Landry Tiacoh, André Offianan Touré, Ronald Perraut, Joseph Djaman, Sylviane Pied

**Affiliations:** 1 Département de Parasitologie-Mycologie, Institut Pasteur de Côte d’Ivoire, Abidjan, Côte d’Ivoire; 2 UMRS959, Sorbonne Université, INSERM, Immunology-Immunopathology-Immunotherapy (i3), 75005 Paris, France; 3 IGF, Univ Montpellier, CNRS, Inserm, Montpellier, France; 4 Centre Pasteur Garoua, Garoua, Cameroon; 5 UFR Biosciences, Université FHB Cocody, Abidjan, Côte d’Ivoire; 6 Univ. Lille, CNRS, Inserm, CHU Lille, Institut Pasteur de Lille, U1019, UMR 8204, CIIL - Centre d’Infection Et d’Immunité de Lille, 1 Rue du Professeur Calmette, 59000 Lille, France

**Keywords:** Malaria, *Plasmodium falciparum*, Autoantibody, Asymptomatic malaria, Immunity

## Abstract

**Background:**

Naturally acquired immunity to *Plasmodium falciparum* involves parasite-specific antibodies as well as autoreactive responses that may contribute to immune regulation and tolerance. Characterizing these antibody repertoires across clinical phenotypes and transmission settings may help distinguish asymptomatic carriage from clinical malaria and identify biomarkers of exposure and protection.

**Methods:**

We conducted a cross-sectional study in three malaria-endemic settings in Côte d’Ivoire (Korhogo, Man, and Abobo). Individuals with mild malaria (MM; *n* = 94) were enrolled across all three sites, whereas asymptomatic carriers (AS; *n* = 33) and endemic controls (EC; *n* = 39) were enrolled in Abobo only. Total IgG responses to *P. falciparum* (3D7 whole-parasite extract), self-antigens (human brain extract), and mosquito exposure (gSG6-P1) were measured by ELISA or Luminex. The breadth and specificity of autoreactive IgGs were assessed by PANAMA blotting followed by mass spectrometry. Multivariate analyses were used to identify antigenic signatures associated with clinical phenotype and, among MM individuals, differences in transmission intensity.

**Results:**

Compared with MM, AS individuals exhibited lower total IgG reactivity to *P. falciparum* antigens but higher levels of autoreactive IgG3 with a broader antigenic repertoire. Proteomic analysis identified γ- and β-actin, as well as several metabolic enzymes (including succinate-CoA ligase, acetyl-CoA acyltransferase 2, acyl-CoA dehydrogenase, and glutaryl-CoA dehydrogenase) as targets distinguishing AS and EC from MM. Among MM cases, a cluster of brain antigens in the 50–75 kDa range differentiated sites with distinct transmission intensities. IgG responses to gSG6-P1 correlated with autoreactive IgG levels, suggesting an association between mosquito exposure and autoreactive immune responses.

**Conclusions:**

Asymptomatic *P. falciparum* infection in a high-transmission setting was associated with enhanced autoreactive IgG3 responses, a profile that may reflect clinical tolerance and cumulative exposure. Autoreactive antibody signatures, together with markers of mosquito exposure, may serve as candidate biomarkers of malaria exposure and naturally acquired immunity and could inform surveillance and control strategies in endemic areas.

**Graphical Abstract:**

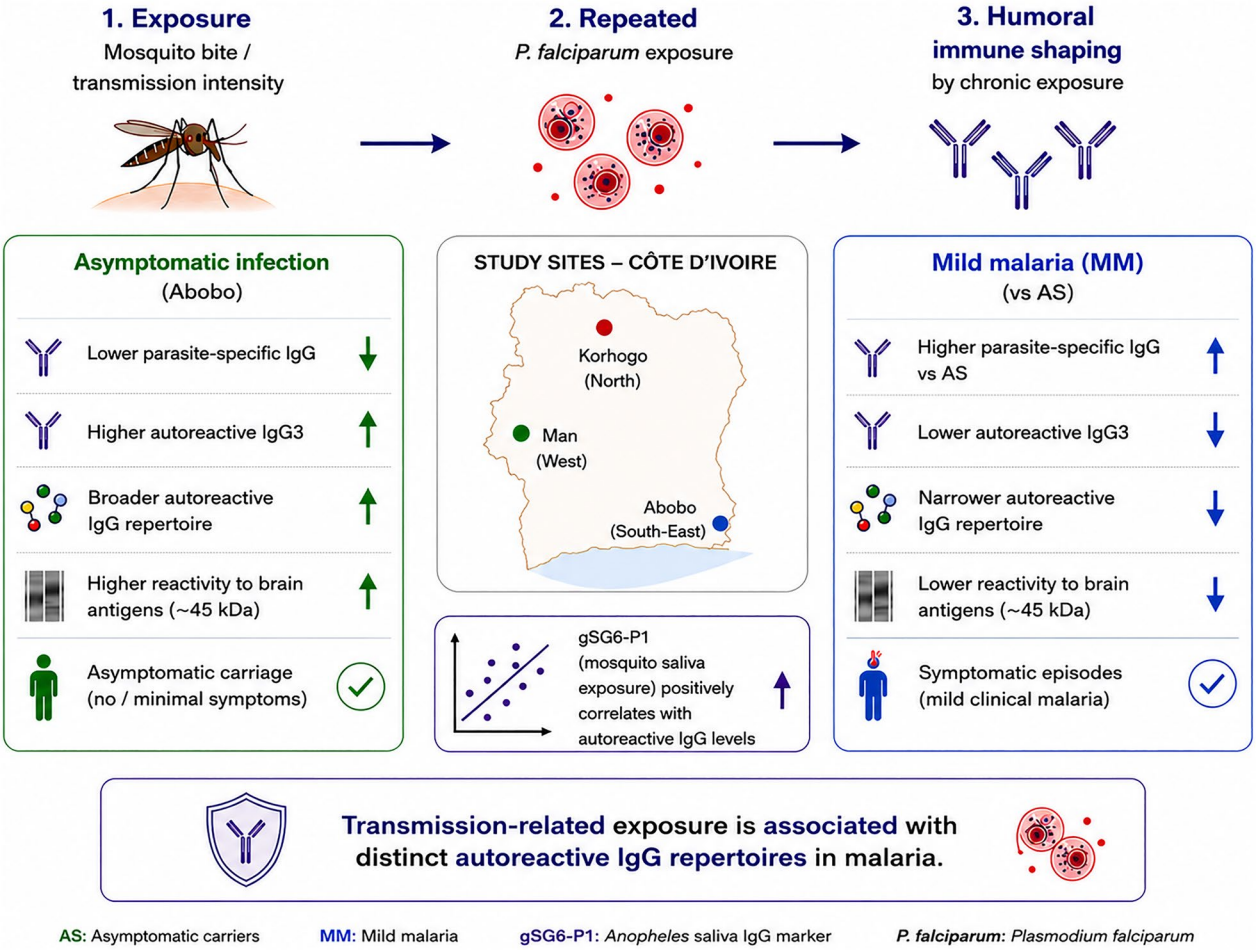

**Supplementary Information:**

The online version contains supplementary material available at 10.1186/s13071-026-07508-9.

## Background

Despite significant global efforts toward eradication, malaria caused by *Plasmodium* spp. remains a major public health burden in sub-Saharan Africa. According to the WHO World malaria report 2025, an estimated 282 million malaria cases and 610,000 malaria deaths occurred worldwide in 2024, with the WHO African Region accounting for approximately 95% of both cases and deaths. This high burden is compounded by challenges such as the lingering impacts of COVID-19 on health services, ongoing conflicts, and limited access to care [1].

Cerebral malaria (CM) is a leading cause of malaria-related mortality, especially in children under 5 years of age and immunologically naïve or immunocompromised adults [1]. In contrast, individuals living in endemic areas often acquire clinical immunity, a non-sterilizing immune response that controls parasitemia without causing symptoms, leading to the development of asymptomatic infections. This state, historically termed premunition by E. Sergent, emerges after repeated exposure over several years [2, 3]. However, the acquisition and maintenance of asymptomatic malaria vary depending on transmission intensity [4–6], and the precise immunological mechanisms conferring protection remain incompletely understood.

Protective immunity during asymptomatic *P. falciparum* infection involves a complex network of CD4⁺ and CD8⁺ T cells, B cells, antibodies, and inflammatory mediators [7, 8]. The parasite’s multiple life stages further complicate immune targeting, as each stage requires distinct immunological strategies [9]. Humoral immunity, particularly through parasite-specific IgG, plays a key role in parasite clearance. This is exemplified by passive-transfer studies showing that IgG from malaria-immune adults can reduce parasitemia and improve clinical outcomes in non-immune patients with *P. falciparum malaria* [10, 11]. Antibodies targeting free parasite stages, sporozoites and merozoites, can block invasion of hepatocytes and erythrocytes, promote phagocytosis via Fc receptor binding, and activate the complement cascade [12–18]. However, the exact in vivo mechanisms remain under investigation.

In addition to parasite-specific responses, *P. falciparum* infection also induces polyclonal B-cell activation, leading to elevated levels of total IgG and the emergence of autoantibodies. These include antibodies against phospholipids, cardiolipin, single- and double-stranded DNA, and rheumatoid factors [19–22]. Interestingly, although these autoantibodies (AAbs) are frequently observed in asymptomatic individuals, they do not typically lead to autoimmune disease, suggesting a potentially regulatory or protective function [23–25].

The extent to which self-reactive and parasite-specific antibodies contribute to protection against symptomatic malaria remains unclear. Moreover, few studies have investigated how transmission intensity and parasite genetic diversity influence these antibody repertoires. Some evidence suggests that increased IgG autoreactivity may result from molecular mimicry between host and parasite antigens, particularly in high-diversity settings [26]. A comparative study in Côte d’Ivoire and Gabon revealed region-specific differences in the genetic diversity of *P. falciparum* merozoite genes, which may shape both epidemiological and immunological profiles [27]. In high-transmission settings, frequent superinfection and recombination sustain extensive antigenic diversity, whereas lower transmission areas are characterized by more limited and structured parasite populations. Such differences are likely to shape both malaria epidemiology and host immune responses, including the breadth and durability of antibody repertoires.

In this study, we investigated the antibody repertoires of *P. falciparum*-infected individuals from three transmission settings in Côte d’Ivoire. We focused on the relationship between autoantibody profiles and parasite-specific IgG responses in asymptomatic carriers, and examined how transmission intensity influences the expression of self-reactive antibodies within symptomatic malaria cases.

## Methods

### Study population

Biological samples were collected from participants enrolled at three malaria sentinel sites in Côte d’Ivoire (Korhogo, Man, and Abobo) representing distinct ecological zones. Recruitment took place between September and November 2013, following the rainy season corresponding to peak malaria transmission [28]. These localities were chosen because they are part of the sentinel sites of the NMCP and, therefore, included in the malaria epidemiological surveillance program.

Korhogo (9°53′N, 6°49′W), located in the northern savannah bordering Mali and Burkina Faso, has a Sudanian climate with malaria transmission lasting 6–8 months annually. Man (7°24′N, 7°33′W) lies in the western forested and mountainous region with abundant rainfall (~ 1800 mm/year) and perennial transmission. Abobo, in the southern district of Abidjan, is a coastal periurban area characterized by the presence of the lagoon, where transmission also occurs year-round.

Recent entomological monitoring suggests that malaria transmission was very heterogeneous across Côte d’Ivoire [29]. In Korhogo, the annual entomological inoculation rate (EIR) of 158 infectious bites/person/year is lower than the mountainous West region of Man (303 infectious bites/person/year), where transmission remains intense with multiple vector species involved. No EIR data were available for Abobo. However, health facility morbidity records from 2013 to 2024 confirmed intense transmission in this area each year. All the sera collected from participants were stored at − 80 °C until immunological analysis.

### Definition of malaria patient groups

Malaria cases were classified according to World Health Organization (WHO) criteria for uncomplicated malaria. Mild malaria (MM) was defined by the presence of fever and a positive thin blood smear for *Plasmodium falciparum*. Individuals with a positive blood smear but no clinical symptoms were classified as asymptomatic malaria (AS) cases. Children with neither clinical symptoms nor detectable parasitemia on thin or thick blood smears were enrolled as endemic controls (EC). In total, 94 patients with mild malaria were recruited from health centers in Abobo (Formation Sanitaire Anonkoua-Kouté), Korhogo (Centre de Santé Petit Paris), and Man (Centre de Santé Urbain de Libreville). Additionally, 33 asymptomatic schoolchildren from Abobo were selected during a cross-sectional survey conducted near the Anonkoua-Kouté health facility. Malaria diagnosis included rapid diagnostic testing (RDT), blood collection for biological analysis, and microscopic examination of thick blood smears for parasite quantification. The RDT used was Bioline^™^ Malaria Ag P.f (HRP2/pLDH) (Abbott Diagnostics Korea Inc), detecting PfHRP2 and pLDH of *P. falciparum* in human whole blood, and was performed according to the manufacturer’s instructions. All participants were investigated using both malaria rapid diagnostic testing and microscopy. For microscopy, both thick and thin blood smears were prepared for each participant; thick smears were used for parasite detection and quantification, whereas thin smears were used for species confirmation. Group classification was based on clinical status and microscopy-confirmed *P. falciparum* infection, with RDT used as an additional diagnostic screening tool. Parasite densities were determined by counting the number of parasites per 200 leukocytes (or per 500, if the count was less than 10 parasites per 200 leukocytes), assuming a leukocyte count of 8000 cells/µl. Asexual parasitemia of any level was reported as positive, and a smear was considered negative after reviewing 100 high-powered fields. All smears were examined by two independent expert microscopists at the reference laboratory. A third expert microscopist resolved any discrepancies arising from the results of the two microscopists.

### Determination of total plasma IgG and IgM levels

Total IgG and IgM levels in plasma were measured using a sandwich ELISA. Flat-bottomed 96-well plates (NUNC, MaxiSorp) were coated overnight at 4 °C with monoclonal antibodies specific for human IgG or IgM (5 µg/mL) in PBS. Plates were then washed three times with PBS containing 0.1% Tween 20 (PBST), and blocked for 1 h at 37 °C with PBS containing 1% gelatin.

Plasma samples were diluted 1:100 in PBS supplemented with 1% gelatin and 0.1% Tween 20, and added to the wells in duplicate. After 1 h of incubation at 37 °C, plates were washed five times with PBST, then incubated for another hour at 37 °C with peroxidase-conjugated anti-human IgG or IgM antibodies (1:5000 in PBS with 1% gelatin and 0.1% Tween 20).

Color development was carried out using the substrate 2,2′-azino-bis(3-ethylbenzthiazoline-6-sulfonic acid) (ABTS) for 1 h at room temperature. Optical density (OD) was measured at 405 nm using an Emax ELISA plate reader. Total IgG and IgM concentrations were determined from assay standard curves and expressed in g/L. Antigen-specific ELISA responses were expressed as OD values or OD ratios normalized to the malaria-naïve control pool, as indicated in the corresponding analyses.

### Quantification of total IgG and subclasses (IgG1-4) against *Plasmodium falciparum* and human brain antigens

Total plasma IgG specific to *Plasmodium falciparum* or human brain antigens was quantified by enzyme-linked immunosorbent assay (ELISA). Ninety-six-well plates (NUNC, MaxiSorp) were coated overnight at 4 °C with 1 μg/mL of either *P. falciparum* 3D7 schizont-stage soluble protein extract or human brain protein extract. Plates were then blocked with 1% gelatin in PBS. Patient plasma samples were diluted respectively for IgG detection (1:100 for parasite extract and 1:50 for brain extract), and for subclasses IgG1-4 (1:50 for parasite extract and 1:25 for brain extract). One hundred microliters of diluted plasma were added in duplicate to each well and incubated for 1 h at 37 °C. After washing, plates were incubated with biotin-conjugated mouse anti-human IgG antibody (BD Pharmingen, San Diego, USA) at a dilution of 1:2000, followed by incubation with peroxidase-conjugated streptavidin. IgG subclasses were assessed using biotinylated mouse monoclonal antibodies specific for human IgG subclasses, anti-human IgG1 (1:4000 dilution; SB 59052–08, Bedfordshire, UK), anti-human IgG2 (1:3000 dilution; 555874, BD, Germany), anti-human IgG3 (1:4000 dilution; 59210-08, Sigma, St. Louis, USA), and anti-human IgG4 (1:3000 dilution; 555884, BD, USA). Detection was performed using 2, 2′-azino-bis (3-ethylbenzthiazoline-6-sulfonic acid) (ABTS; Sigma-Aldrich, France) as substrate. The colorimetric reaction was allowed to proceed for 30 min (streptavidin incubation) and 1 h (substrate development), and optical density (OD) was read at 405 nm. For each assay, a cut-off value was established using a pool of plasma from European women living in northern France (Lille) with no history of *P. falciparum* exposure.

Inter-plate assay homogeneity was assessed using two positive controls: a pool of hyperimmune sera (Ivorian pool) and a pool of immune IgG (IgMH pool, kindly provided by Professor Marcel Hommel) in all the plates. Assay validation required that inter-plate variation did not exceed 15%. In addition, the standardization procedure incorporated a predefined sample distribution strategy to avoid systematic plate effects, ensuring that samples of the same type (e.g., originating from the same clinical phenotype or the same village) were not analyzed exclusively on a single plate.

### Detection of IgG responses to *Anopheles gambiae* salivary antigen (gSG6-P1)

IgG responses to the *Anopheles gambiae* salivary peptide gSG6-P1 were measured using a multiplex bead-based assay (MBA), as previously described [30, 31]. The assay relied on covalent coupling of gSG6-P1 peptide, conjugated to bovine serum albumin (BSA), onto carboxylated magnetic Luminex beads.

Each assay included a pool of sera from malaria-immune adults from Dielmo (Senegal) as a positive control, and a pool of European and African non-immune sera as negative and normalization controls. IgG levels were expressed as mean fluorescence intensity (MFI) or log-transformed MFI values. The positivity threshold was defined as the mean net signal of the naïve controls plus three standard deviations (mean + 3 SD).

### Self-reactive antibody profiling using the PANAMA-blot method

The PANAMA-Blot method was used to assess the antibody repertoire directed against brain antigens, as previously described [24, 32]. This technique enables the global profiling of self-reactive IgG responses across a broad panel of tissue-derived antigens. Human brain extract was used as a complex source of self-antigens and does not imply brain-specific autoimmunity, as the majority of proteins detected are ubiquitously expressed across multiple tissues.

Briefly, proteins from normal human brain tissue, obtained post-mortem from a healthy volunteer donor (Novus^®^), were separated by SDS-PAGE (10% acrylamide) at 25 mA. The proteins were then transferred onto nitrocellulose membranes (Schleicher & Schüll, Dassel, Germany) by semi-dry electrotransfer (Pasteur Institute, Paris) for one hour at 0.8 mA/cm^2^. Membranes were blocked overnight at room temperature using PBS supplemented with 0.2% Tween 20 (PBST), and subsequently incubated with individual plasma samples, each adjusted to a final IgG concentration of 200 μg/mL, using the Miniblot Cassette System (Immunetics, MA, USA). Incubations were performed for four hours at room temperature with gentle agitation.

Detection of bound IgG was performed using rabbit anti-human IgG conjugated to alkaline phosphatase (Sigma-Aldrich, France), followed by development with NBT/BCIP (nitro blue tetrazolium/5-bromo-4-chloro-3-indolyl-phosphate; Promega, France) for 90 min at room temperature. Immunoreactivity was visualized and quantified, and signal intensities were normalized using IGOR software (version 3.16; Wavemetrics, Lake Oswego, OR, USA). A pooled standard plasma sample, composed of diverse sera of Ivorian individuals diluted 1:20, was included in duplicate on each membrane for inter-blot normalization and intensity adjustment.

### Protein identification by mass spectrometry

Briefly, human brain extract was separated by 10% SDS-PAGE. After Coomassie staining, the band corresponding to Sect.  11, which exhibited the strongest immunoreactivity, was excised for analysis by peptide mass fingerprinting. Gel bands were collected using the ProPic Investigator system (Genomic Solutions, Ann Arbor, MI, USA) and transferred into a 96-well plate. Destaining, reduction, alkylation, and in-gel digestion with trypsin were carried out using the ProGest Investigator platform (Genomic Solutions, Ann Arbor, MI, USA), followed by peptide extraction. After desalting with C18-μZipTip columns (Millipore), peptides were directly eluted using the ProMS Investigator system (Genomic Solutions, Ann Arbor, MI, USA) onto a 96-well stainless-steel MALDI target plate (Applied Biosystems/MDS SCIEX, Framingham, MA, USA) in 0.5 μL of CHCA matrix solution (5 mg/mL in 70% acetonitrile, 30% H_2_O, 0.1% trifluoroacetic acid). MS/MS spectra were interpreted using the Mascot search engine (version 2.4.0, Matrix Science, London, UK), installed on a local server. Database searches were performed against a composite target-decoy database (40,582 total entries), constructed from the Human Swiss-Prot database (TaxID = 9606, 20,173 entries), supplemented with sequences for recombinant trypsin and common laboratory contaminants (117 entries). The mass tolerance was set at 0.2 Da for both precursor and fragment ions.

### Statistical analysis

Group comparisons for quantitative variables were performed using non-parametric tests: the Mann–Whitney *U* test for two-group comparisons and the Kruskal–Wallis test for comparisons involving more than two groups. Associations between categorical variables were assessed using the Chi-square test. Correlations between continuous variables were evaluated using Spearman’s rank correlation coefficient. All tests were two-tailed, and p-values below 0.05 were considered statistically significant.

Immunoblot data were analyzed using IGOR software (Wavemetrics, Lake Oswego, OR), including custom-written modules for reactivity profiling. The standard molecular weight scale was divided into discrete sections based on local peaks of immunoreactivity. Absorbance values within these sections were subjected to principal component analysis (PCA), using covariance matrices to identify major patterns of variation across individuals.

All statistical analyses and data visualizations were performed using R software.

## Results

### Demographic characteristics of the study population

Participant characteristics are summarized in Table 1 for the Abobo clinical groups and in Table 2 for symptomatic MM cases recruited across the three sentinel sites. Among MM cases, parasitemia levels measured on day 0 (day of hospital admission) were highly variable across study sites, with significantly lower parasitemia observed in Korhogo compared to the other settings (*p* < 0.01; Table 2). In contrast, the median age of participants did not differ significantly between the three sites, and age distributions were comparable across groups. All three study areas are characterized by variable malaria transmission levels. In the rural setting of Korhogo, the entomological inoculation rate (EIR) is estimated at approximately twofold lower than Man locality, while in Abobo, the prevalence of clinical malaria is more than 2.5 times higher than in the rural sites. Despite widespread use of bed nets, individuals recruited from the urban site (Abobo) experienced high-parasitemia clinical episodes. In Abobo, 10% and 30% of patients exhibited parasitemia levels of < 10,000 and > 100,000 trophozoites per microliter, respectively. In comparison, the corresponding proportions were 26% and 16% in Man, and 56% and 0% in Korhogo, respectively.

**Table 1:** Total IgM, IgG, and parasite density in different clinical groups of Abobo

Groups	MM	AS	EC	*p*-value
Number of patients	31	33	39	
Age median (min–max)	10 (5–15)	10 (6–13)	12 (9–14)	0.6280
Parasite density (parasites/µL) median	46200	810.5	0	< 0.001*
Total IgM (g/L) mean ± SD	0.55 ± 0.89	0.46 ± 0.22	0.49 ± 0.36	< 0.01**
Total IgG (g/L) mean ± SD	13.64 ± 5.42	22.16 ± 8.31	16.23 ± 8.57	< 0.001***

*MM* mild malaria, *AS* asymptomatic carriers, *EC* endemic controls. * Parasite density was compared between MM and AS using the Mann–Whitney U test. ** Total IgM was higher in MM than in AS and EC. *** Total IgG was higher in AS and EC than in MM

**Table 2:** Epidemiological and serological characteristics of symptomatic mild malaria patients recruited in three malaria sentinel sites

Sites	Abobo (periurban)	Korhogo (rural)	Man (rural)
Number of MM patients	31	32	31
Age			
Age < 5 years, n	3	7	10
Age median (range)	10 (3–54)	10.5 (1-70)	10 (1–59)
Parasite density			
Median*	46200	10000	37409
Range	(3480–174000)	(2030–92800)	(2470–120000)
Use of ITN	27%	10%	20%
Malaria clinical incidence rate	232/1000	92/1000	75.5/1000
CI/CI national	247%	98%	80%
Total IgM (g/L)	0.55	0.41	0.47
Total IgG (g/L)	15.54 ± 5.52	15.49 ± 5.586	23.63 ± 6.994
Total *P. falciparum*-specific IgG (OD) mean ± SD	0.82 ± 1.12	1.77 ± 0.75	0.87 ± 1.01

*ITN* Insecticide-treated bed net, *CI* clinical incidence. *Parasite density was higher in Abobo than in Korhogo and Man, with a *p*-value < 0.01. All individuals included in Table 2 were symptomatic mild malaria cases. Asymptomatic carriers and endemic controls were recruited only in Abobo and are described in Table 1

### Total, parasite-specific, and brain-reactive IgG and IgM responses across clinical groups and sites

To characterize systemic and antigen-specific humoral responses across clinical presentations and transmission sites, we measured total IgG and IgM, *Plasmodium falciparum*-specific IgG, and brain-reactive IgG, along with IgG subclasses, in endemic controls (EC), asymptomatic carriers (AS), and mild malaria patients (MM) from Abobo, Korhogo, and Man.

**Fig. 1:**
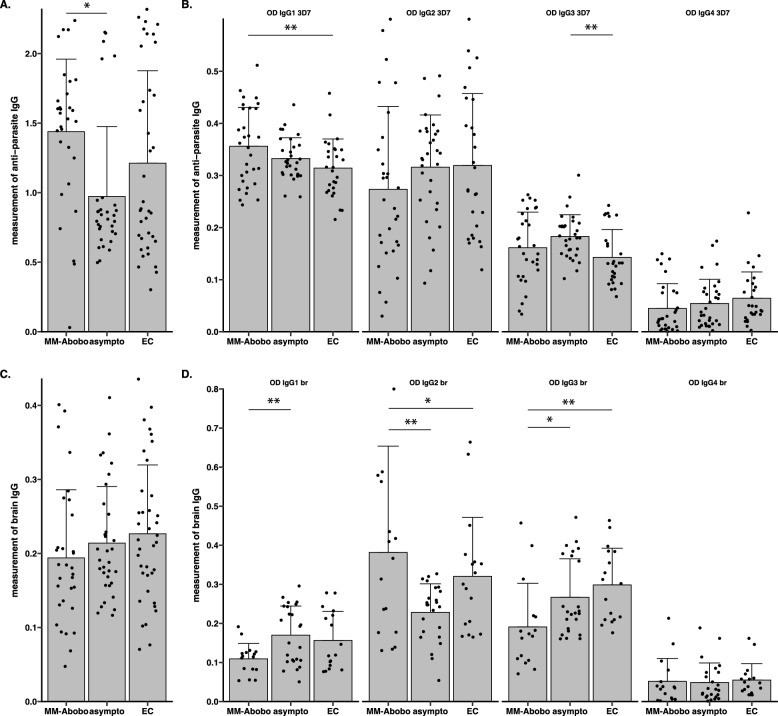
IgG and subclass patterns against brain and parasite extract antigens in different malaria groups of Abobo. Antibody responses, expressed as OD (Optical density), are plotted as a box plot. **A** Antibody IgG response against total parasite antigens, **B** distribution of the subclasses (IgG1, IgG2, IgG3, and IgG4) against parasite antigens. **C** IgG response against brain antigen in malaria groups. **D** Subclasses (IgG1, IgG2, IgG3, and IgG4) against brain antigens in malaria groups. MM: mild malaria; AS: Asymptomatic carrier; EC: endemic control. Asterisks indicate statistically significant differences between groups (* *p* < 0.05; ** *p* < 0.01; *** *p* < 0.001)

In Abobo, 33 AS, 31 MM, and 39 EC individuals were included, with no significant differences in sex or age. Parasitemia was significantly lower in AS than in MM (*p* < 0.001) (Table 1). Total IgM levels at day 0 were significantly higher in MM than in AS and EC. Total IgG levels were higher in AS and EC than in MM in Abobo (*p* < 0.001) (Table 1). In contrast, MM individuals from Man displayed the highest total IgG levels across all sites (Table 2). Total IgG was positively correlated with age (*R* = 0.23, *p* = 0.01). *P. falciparum-specific* IgG was higher in MM than in AS or EC in Abobo (*p* = 0.021; Fig. 1A), with no serological difference between MM groups across study sites (Table 2). A positive correlation with age was observed (*R* = 0.40, *p* = 0.001), but no association was found with parasitemia. Brain-specific IgG levels were comparable across clinical phenotype groups and sites (Fig. 1C). The analysis of IgG subclass responses to parasite antigens revealed distinct patterns according to clinical presentation. A significant difference in anti-parasite IgG1 levels was observed between patients with malaria (MM) and endemic controls (EC) (*p* = 0.008). In addition, anti-parasite IgG3 levels differed significantly between endemic controls and asymptomatic carriers (*p* = 0.007). IgG2 anti-parasite levels tended to be higher in asymptomatic individuals than in MM patients, without reaching statistical significance (Fig. 1B), whereas IgG2 anti-brain antigen levels were significantly higher in patients with mild malaria (MM) compared with asymptomatic individuals and endemic controls (EC). However, IgG subclass analysis revealed a predominant IgG3 response to brain antigens in AS and EC compared to MM (*p* = 0.024) (Fig. 1D).

These data show that asymptomatic individuals maintain strong total IgG levels and exhibit elevated IgG3 responses to brain antigens, while displaying lower P. falciparum-specific IgG than MM individuals in Abobo.

### Asymptomatic individuals show broader and more intense self-reactive IgG responses to brain antigens

**Fig. 2:**
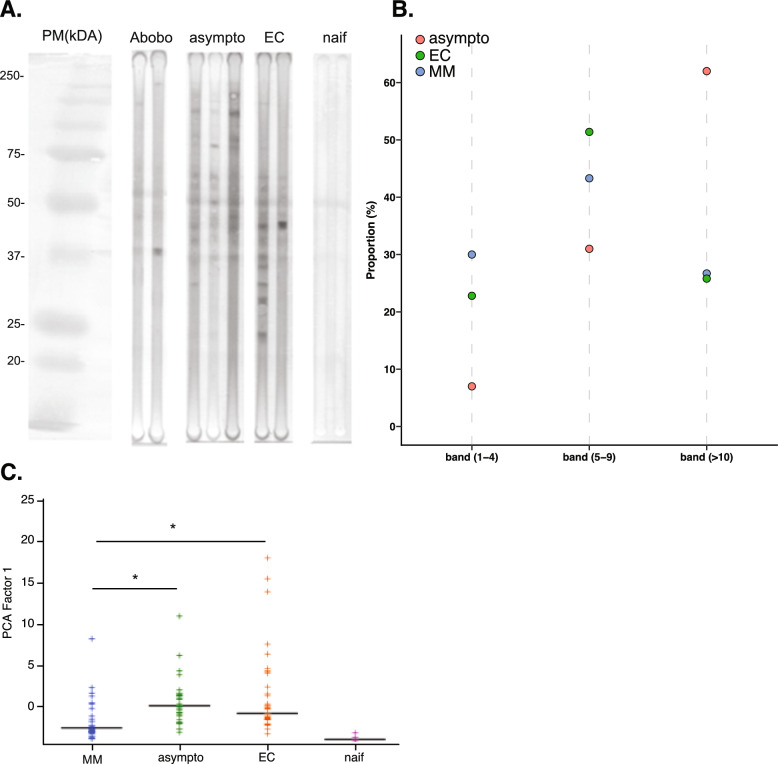
Profiles of IgG reactivities to brain antigens in the different participant groups from Abobo. **A** Example immunoblot profiles of sera reactive to brain antigens from EC, AS, and MM participants. Each strip contains approximately 10 µg of human brain extract; MW, molecular-weight marker; malaria-naïve, European control sera from individuals with no history of malaria exposure. **B** Percentage of individuals in each clinical group distributed according to the number of reactive bands (1–4, 5–9, and > 10). **C** PCA factor 1 score from adjusted IgG reactivity profiles. Asterisks indicate statistically significant differences between groups (* *p* < 0.05; ** *p* < 0.01; *** *p* < 0.001)

To assess the diversity and intensity of self-reactive IgG responses during *P. falciparum* infection, we measured IgG reactivity to human brain protein extracts by quantitative immunoblot in EC, AS, and MM individuals from Abobo (Fig. 2A).

Immunoblot profiles were divided into 21 defined sections based on reference serum reactivity (Figure S1). All malaria-exposed groups and endemic controls (AS, MM, and EC) displayed a high number of reactive bands compared with malaria-naïve European individuals (Fig. 2A). Moreover, these malaria groups showed increased self-reactive IgG, with AS individuals displaying the broadest response: 62% recognized more than 10 sections, compared to 26.7% in MM and 25.8% in EC (*p* < 0.01) (Fig. 2B).

Single linkage hierarchical clustering identified two reactivity zones (Sects. 0–5 and 6–16) present in AS and EC, but markedly reduced in MM. Principal component analysis (PCA) showed higher PCA-1 scores in AS than in MM (*p* < 0.01; Fig. 2C), indicating greater total reactivity. PCA-1 correlated positively with brain-specific IgG levels (*R* = 0.45, *p* < 0.001). Anti-brain reactivity (PCA-1 score) was also significantly correlated with age (Spearman *R* =  + 0.29, *p* < 0.01; Figure S2) but not with sex or parasitemia. These results demonstrate that asymptomatic individuals mount a broader and more intense self-reactive IgG response than both controls and symptomatic patients, suggesting an expanded autoreactive repertoire associated with asymptomatic infection.

### A 45 kDa brain antigen (Sect.  11) is a dominant target of self-reactive IgG in asymptomatic individuals

To identify specific self-antigens driving the expanded autoreactive IgG response, we analyzed section-specific reactivity profiles from the brain immunoblot of all participant groups together.

**Fig. 3:**
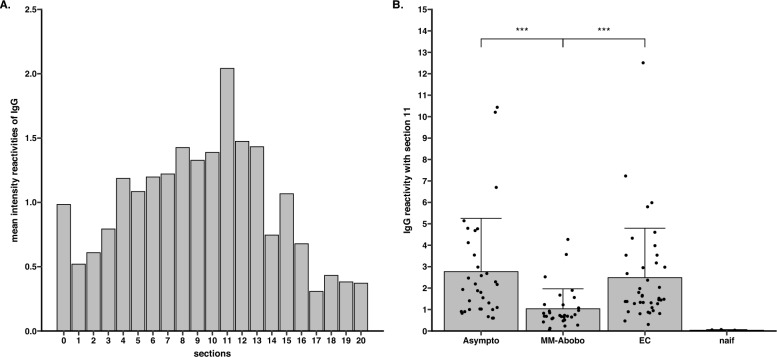
Patterns of brain self-reactive IgG responses in AS, MM & EC. **A** Distribution of mean intensity reactivities of IgG from AS, MM & EC recognized sections in brain extract. **B** IgG reactivity level against Sect.  11 in AS, EC, and MM groups. Asterisk indicates a significant difference in antibody reactivity level (* *p *< 0.05; ** *p* < 0.01; *** *p* < 0.001)

Among the 21 sections, Sect.  11, corresponding to a protein band of approximately 45 kDa, was a specific signature of reactivity in all groups and was also the most strongly and frequently recognized in AS individuals, as shown by PCA factor 1 predominantly driven by IgG reactivity to Sect.  11 **(**Fig. 3A).

In accordance with the results of the PCA, Sect.  11 was recognized by IgG in 84% of asymptomatic individuals, whereas recognition of this section was significantly less frequent in individuals with mild malaria (*p* < 0.01) (Fig. 3B). Although we also observed high variability across all bands, no particular signature of reactivity could be identified in MM patients.

Together, these analyses identify band or Sect.  11 as a dominant autoreactive target in asymptomatic malaria and demonstrate that its high prevalence and strong contribution to PCA factor 1 underlie the broader autoreactive IgG profile observed in this group.

### The dominant brain antigen recognized in asymptomatic individuals contains cytoskeletal and metabolic proteins

**Fig. 4:**
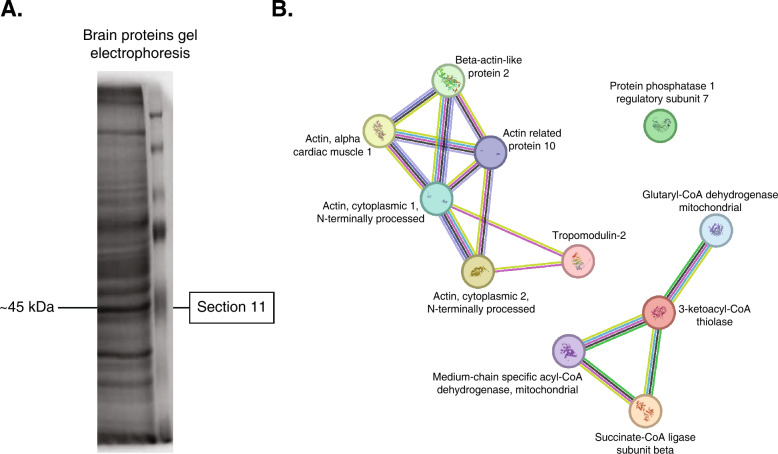
Identification and characterization of proteins contained in Sect.  11. **A** Protein identification by mass spectrometry analysis of the ~ 45 kDa band corresponding to Sect.  11. Eleven human proteins were identified with a spectral abundance ratio greater than 60%, as described in the Materials and Methods section. **B** Protein–protein interaction network of the proteins identified in Sect.  11, highlighting cytoskeletal and metabolic clusters. Network analysis was performed using STRING (Search Tool for the Retrieval of Interacting Genes/Proteins)

To characterize the molecular composition of the dominant self-antigenic region identified in Sect.  11 (Fig. 4A**)**, we performed mass spectrometry analysis of the corresponding 45 kDa brain protein band.

A total of 11 proteins were identified with more than 60% spectral abundance. These included cytoskeletal components such as β-actin and γ-actin, and metabolic enzymes involved in mitochondrial lipid metabolism, including succinate-CoA ligase, acetyl-CoA acyltransferase 2, acyl-CoA dehydrogenase, and glutaryl-CoA dehydrogenase. Protein–protein interaction analysis using STRING revealed two major networks (Fig. 4B): a cytoskeletal cluster related to membrane architecture and cell motility, and a catalytic cluster linked to lipid biosynthesis.

These data show that the dominant self-antigenic region recognized in asymptomatic individuals contains conserved host proteins involved in structural and metabolic functions, possibly reflecting targets of molecular mimicry or cross-reactivity with parasite components.

### Self-reactive and parasite-specific IgG responses are differentially distributed across clinical groups

**Fig. 5:**
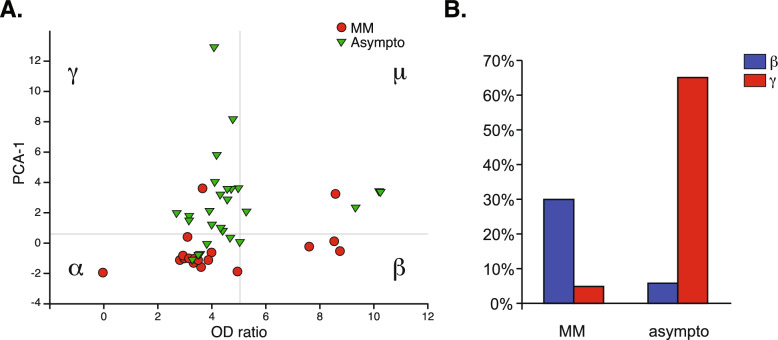
Relationship between auto-antibody response and *Plasmodium falciparum*-specific antibody in AS and MM groups. **A** Scatter plot showing the relationship between brain-reactive IgG (PCA-1 score) and *P. falciparum*-specific IgG (rOD) in AS and MM individuals. Participants were classified into four subgroups using the thresholds PCA-1 = 0.5 and rOD = 5: *α*, low self-reactive (PCA-1 < 0.5) and low *P. falciparum*-specific IgG (rOD < 5); *β*, low self-reactive (PCA-1 < 0.5) and high *P. falciparum*-specific IgG (rOD ≥ 5); *γ*, high self-reactive (PCA-1 ≥ 0.5) and low *P. falciparum*-specific IgG (rOD < 5); *μ*, high self-reactive (PCA-1 ≥ 0.5) and high *P. falciparum*-specific IgG (rOD ≥ 5). **B** Proportions of individuals in the β and γ subgroups in AS and MM

To explore how self-reactive and parasite-specific antibody responses co-distribute in individuals with different clinical outcomes, we classified participants into four subgroups based on defined thresholds for brain-specific IgG (PCA-1 = 0.5) and *P. falciparum*-specific IgG (rOD = 5) (Fig. 5A). The subgroups were defined as follows: individuals with low self-reactive and low parasite-specific IgG (*α*), low self-reactive and high parasite-specific IgG (*β*), high self-reactive and low parasite-specific IgG (*γ*), and high levels of both antibody types (*μ*).

Asymptomatic individuals (AS) were largely concentrated in subgroup *γ*, with 65% exhibiting high autoreactive but low parasite-specific IgG levels. In contrast, mild malaria patients (MM) were predominantly found in subgroup *β*, with 30% showing the opposite profile–low self-reactivity and high parasite-specific responses. These distributions were significantly different between groups (*p* < 0.001 for *γ*; *p* < 0.01 for *β*) (Fig. 5B). The *μ* subgroup, characterized by high levels of both responses, included a more balanced representation of participants.

These results reveal that asymptomatic and symptomatic individuals are associated with distinct immunoglobulin response profiles, with AS favoring self-reactive dominance and MM characterized by parasite-specific responses.

### Transmission intensity and mosquito exposure influence the magnitude and pattern of self-reactive IgG responses

To assess whether malaria transmission intensity modulates the autoreactive IgG repertoire, we compared brain-specific IgG responses across the three sentinel sites: Abobo (peri-urban), Man and Korhogo (rural settings).

**Fig. 6:**
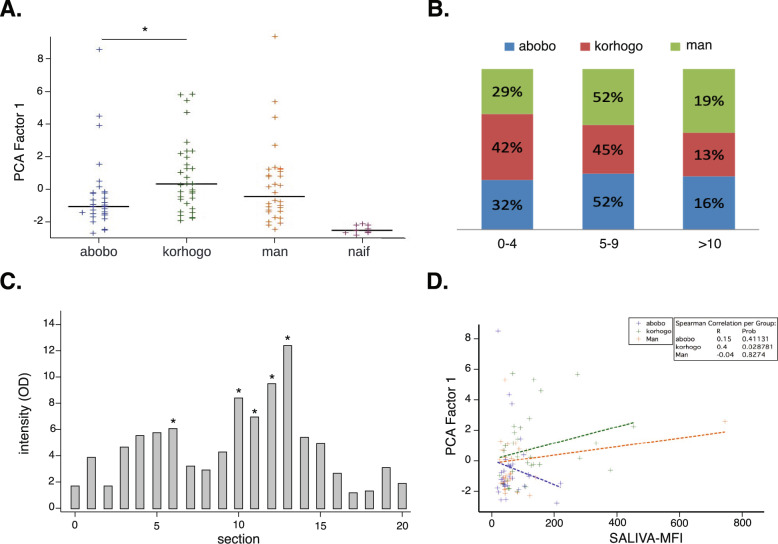
Pattern of brain self-reactive IgG response and correlation with saliva response in the different sentinel sites. **A** PCA factor 1 score from adjusted IgG reactivity profiles in the sentinel sites. Groupwise distribution of PCA factor 1 score. **B** Distribution of brain antigenic bands recognized in Abobo, Korhogo, and Man. **C** Mean OD intensity by section; asterisks indicate sections significantly differing between sites by Kruskal–Wallis test. **D** Relationship between self-reactive IgG and saliva IgG response according to the study site

Low-level reactivity (0–4 bands) was observed across all three sites and was highest in Korhogo, whereas reactivity to > 10 bands was less frequent in Korhogo than in Abobo and Man (Fig. 6B). Principal component analysis of anti-brain IgG profiles revealed that PCA-1 scores, adjusted for total IgG concentration (1 mg/mL), were significantly higher in Korhogo compared to Abobo and Man (*p* < 0.01; Fig. 6A). Korhogo also reported the lowest usage of vector control measures, such as bed nets (Table 2), suggesting increased exposure to mosquito bites. PCA-1 scores were positively correlated with age (Spearman *R* = 0.45, *p* < 0.001) and total plasma IgG concentrations (Spearman *R* = 0.38, *p* < 0.001), but showed no association with parasitemia or sex. Differential analysis of specific brain protein bands identified sections S6, S10, S11, S12, and S13 as significantly discriminating the sites in terms of autoreactive IgG reactivity (Fig. 6C). Given the known link between mosquito exposure and IgG responses to salivary proteins, we measured IgG levels against the *Anopheles* gSG6-P1 peptide by Luminex assay. These levels were highest in Korhogo and showed a positive correlation with PCA-1 scores across all individuals (*R* = 0.27, *p *< 0.05; Fig. 6D), reinforcing the connection between vector exposure and the intensity of self-reactive antibody responses.

Together, these findings indicate that both transmission intensity and mosquito exposure shape the autoreactive IgG landscape in malaria-endemic populations, with higher exposure being associated with broader and stronger self-reactive immune profiles.

## Discussion

### Asymptomatic malaria and immunity in high-transmission settings

In Côte d’Ivoire, the distribution of asymptomatic malaria (AS) is heterogeneous and closely influenced by age and transmission intensity. AS individuals are considered a major reservoir for silent transmission and represent the majority of infections in high-transmission regions, where they also constitute a valuable model of naturally acquired clinical protection. The country maintains a network of 12 sentinel sites dedicated to molecular surveillance of drug resistance and immuno-epidemiological monitoring to inform national malaria control strategies. One such site is Abobo, a peri-urban area in the south, where year-round transmission is intense. In this setting, young children often carry high parasite densities in the absence of clinical symptoms [33], suggesting the development of naturally acquired immune mechanisms that confer protection against blood-stage malaria [34]. Continuous exposure in such contexts favors the rapid acquisition of premunition, largely driven by antibody-mediated immune responses.

In this study, we aimed to characterize the repertoire of both parasite-specific and self-reactive antibodies in individuals from diverse clinical groups and epidemiological settings. To achieve this, we applied a global immunoprofiling approach based on quantitative immunoblotting using tissue protein extracts. This method enables the detection and comparison of global antibody reactivity patterns across a wide spectrum of self-antigens but is not designed to resolve epitope-level specificity. Rather, it offers a robust means to assess the overall architecture of humoral responses and their variation between populations.

### Elevated autoreactive antibody levels in asymptomatic individuals

Asymptomatic children displayed elevated levels of autoantibodies compared to those with symptomatic malaria. This autoreactive response was positively correlated with both age and total plasma IgG levels, consistent with previous reports suggesting a link between cumulative exposure, polyclonal B cell activation, and premunition [2, 24, 35]. Prior studies have also described increased antibody responses against both human brain antigens and heterologous bacterial proteins such as those from *Escherichia coli* in asymptomatic individuals, further supporting the notion of broad polyclonal IgG reactivity in this population [36].

### Functional relevance of autoreactive antibodies in malaria immunity

Autoreactive B cells are a natural component of the physiological B cell repertoire [37], and the antibodies they produce can target a wide variety of self-antigens [38]. In the context of malaria, self-reactive antibodies, often polyreactive, may contribute to protective immunity in several ways. For instance, they can interfere with intra-erythrocytic parasite development by binding red blood cell components or by enhancing immune clearance. Elevated levels of autoantibodies have frequently been reported during acute or chronic *Plasmodium* infections, suggesting a potential role in nonspecific parasite elimination through polyclonal B cell activation [39].

This type of activation likely results from repeated waves of antigen exposure and B-cell stimulation throughout infection. A study conducted in Brazil [40] described a population of autoreactive B cells emerging during malaria that expressed CD11c and the transcription factor T-bet. The induction of these cells was shown to involve multiple signaling pathways, including those mediated by the IFN-γ receptor, TLR9, and the B cell receptor, suggesting a coordinated immune program driving autoreactivity.

### Autoreactive and parasite-specific antibody responses are independent in infected individuals

In infected individuals, we observed no significant correlation between parasite-specific and self-reactive antibody responses, indicating that the induction of autoreactive IgG during *Plasmodium falciparum infection* is not directly proportional to parasite-directed humoral immunity. Notably, a candidate self-antigen was selectively detected in individuals with asymptomatic malaria (AS), further supporting the notion that autoreactive responses are differentially regulated according to clinical presentation.

These findings suggest that autoreactive antibody production during malaria is unlikely to arise solely from antigenic cross-reactivity between parasite and host proteins or from nonspecific polyclonal B cell activation. Instead, autoreactive responses may originate from distinct B cell compartments that are selectively expanded or activated during infection. One possibility is the involvement of CD27⁺ marginal zone-like B cells, which have been implicated in the production of autoreactive antibodies in several autoimmune and chronic inflammatory conditions. Alternatively, a fraction of the autoreactive antibodies detected may correspond to so-called “natural antibodies,” which are generated independently of overt antigenic stimulation and are characterized by low affinity and broad polyreactivity [19, 20].

Although the functional relevance of these antibodies remains uncertain, autoreactive responses may contribute indirectly to parasite control by enhancing the clearance of infected or damaged erythrocytes. This hypothesis is consistent with previous observations linking elevated autoreactive antibody levels with lower parasitemia in asymptomatic malaria [24]. Similar mechanisms have been proposed to underlie malaria protection associated with haemoglobinopathies, where altered erythrocyte physiology promotes immune recognition and clearance [41]. Together, these observations support the view that autoreactive antibodies may reflect a regulated component of the immune response associated with clinical tolerance rather than pathological autoimmunity.

### IgG subclass distribution suggests a cytophilic autoreactive profile in asymptomatic individuals

The differential IgG subclass responses observed across clinical groups indicate qualitative differences in humoral immunity associated with malaria outcomes. Significant variations in anti-parasite IgG1 and IgG3 levels between groups suggest that cytophilic antibodies may be modulated by infection status and cumulative exposure. Notably, the elevated IgG2 responses against brain antigens in patients with mild malaria compared with asymptomatic individuals and endemic controls may reflect immune activation associated with tissue inflammation rather than parasite-directed protection. This finding suggests that IgG2 responses to host antigens could be a marker of immunopathological processes occurring during symptomatic infection, while remaining limited in asymptomatic carriage.

More strikingly, we observed significantly elevated IgG3 responses to self-antigens in asymptomatic individuals. IgG3 is a cytophilic subclass known for its potent effector functions, including complement activation and engagement of Fcγ receptors. Its predominance in the autoreactive repertoire of AS individuals suggests a possible contribution to protective immunity. Previous studies have linked IgG3 with enhanced control of intracellular pathogens, including parasites, bacteria, and viruses [42].

Mechanistically, this subclass bias may reflect the underlying polarization of T helper cells. Th1-type responses, characterized by the production of IFN-γ, IL-2, and TNF-α, promote class switching toward IgG1 and IgG3 while suppressing other isotypes. In the context of malaria-associated autoimmunity, as proposed by Moss et al. [43], this Th1 skewing could serve a dual purpose: enhancing classical effector responses while simultaneously favoring autoreactive IgG3 production as an additional tool for immune defense.

### Autoreactive IgG in asymptomatic individuals targeting conserved cytoskeletal and metabolic host proteins

Mass spectrometry analysis of the dominant immunoreactive band (Sect.  11) revealed that autoreactive IgG in asymptomatic individuals primarily targets a set of conserved intracellular proteins. These include cytoskeletal components such as *β*- and *γ*-actin, as well as enzymes involved in mitochondrial lipid metabolism, including succinate-CoA ligase, acetyl-CoA acyltransferase 2, acyl-CoA dehydrogenase, and glutaryl-CoA dehydrogenase. Although initially detected in brain extracts, these antigens are not brain-specific and are ubiquitously expressed in multiple tissues.

Autoreactivity to cytoskeletal elements has previously been described in malaria. In Liberian children living in high-transmission areas, immune responses against neoantigens on red blood cell band 3 proteins were associated with lower parasitemia and functional inhibition of cytoadherence both in vitro and in vivo [44]. Similarly, this fits with the broader concept of physiological autoimmunity and with experimental evidence that *Plasmodium chabaudi* infection in mice induces high levels of IgG autoantibodies [45]. These observations suggest that the autoreactive antibodies detected here may contribute to parasite control by targeting infected or damaged erythrocytes.

Additionally, the enzymes identified in our study are involved in lipid metabolic pathways essential for both host and parasite survival [46–48], and pro-inflammatory conditions may amplify their immunogenicity. Given that these proteins are intracellular, their release into circulation may result from erythrocyte lysis during infection [44, 49], making them accessible to the immune system and potential targets of antibody-mediated clearance.

### Transmission intensity shapes autoreactive IgG profiles in symptomatic malaria

Because asymptomatic individuals and endemic controls were recruited only in Abobo, analysis of the impact of transmission intensity on self-reactive responses was restricted to individuals with mild malaria.

Within this group, and despite substantial interindividual variability across the three study sites, the overall IgG autoreactive signature remained relatively conserved, with a shared core of self-antigens recognized in all settings. However, several specific antigenic bands-namely S6, S10, S11, S12, and S13-discriminated between sites according to transmission intensity, suggesting that certain autoreactive targets vary with epidemiological context.

These bands span both low (S6) and high (S10–S13) molecular weights and may serve as candidate biomarkers for transmission pressure or cumulative exposure. This immunological stratification echoes earlier findings from the same sites, where differences in parasite-specific antigen responses were used to distinguish between endemic areas [28]. The ability of self-reactive antibody profiles to reflect environmental and epidemiological variables positions them as potential complementary tools for population-level surveillance in endemic regions.

### Vector exposure correlates with the intensity of autoreactive IgG responses

To further investigate the factors influencing autoreactive antibody production, we examined the relationship between exposure to mosquito bites and self-reactive IgG responses. IgG levels against the *Anopheles* salivary peptide gSG6-P1 were highest in individuals from Korhogo, the site with the lowest reported use of bed nets based on participant questionnaires. These results are consistent with previous studies conducted in Senegal and the Brazilian Amazon, where elevated anti-gSG6 IgG levels have been linked to increased exposure to malaria vectors and natural immunity acquisition [50, 51].

In our cohort, self-reactive IgG levels were significantly correlated with anti-gSG6 responses, indicating that vector exposure is a contributing factor to the induction or amplification of autoreactive immunity. The gSG6 peptide is a well-established marker of vector exposure and transmission pressure [52, 53], and its correlation with autoreactivity supports the hypothesis that persistent environmental exposure, including to mosquito salivary components, may enhance the breadth and intensity of host-directed immune responses. Additional factors such as nutritional status, host genetic background, co-infections, and other environmental factors may further modulate this effect [54].

Together, these findings underscore the importance of environmental and transmission-related factors in modulating autoreactive immunity and suggest that autoreactive antibody profiles may reflect cumulative exposure rather than pathological autoimmunity.

### Study limitations and perspectives for future investigation

Although this study provides insight into the relationship between parasite-specific and autoreactive antibody responses in malaria-exposed individuals, several limitations should be considered. Because of the cross-sectional design, the observed associations between clinical phenotype, gSG6-P1 responses, and autoreactive IgG profiles cannot establish temporality or causality.

First, the uneven enrollment of clinical groups across sites limits the interpretation of transmission-related effects. Asymptomatic carriers and endemic controls were recruited only in Abobo, restricting comparisons across transmission settings to individuals with mild malaria. While this design allowed us to explore how transmission intensity modulates autoreactive responses among symptomatic infections, it limits broader conclusions regarding transmission effects across all clinical phenotypes.

Second, we did not account for intrinsic host factors, such as genetic polymorphisms, immunodeficiency status, or co-infections, that may influence the development or regulation of autoantibody responses. These variables could modulate both the magnitude and the functional relevance of the observed autoreactivity.

Third, while we identified a correlation between vector exposure and autoreactive IgG levels, the specific immunomodulatory effects of mosquito salivary components on B cell activation and autoantibody production remain poorly understood. Additional studies are needed to dissect the mechanisms by which chronic vector exposure shapes autoreactive humoral immunity.

Fourth, while proteomic analyses identified several host antigens targeted by autoreactive antibodies, the functional properties of these antibodies, including affinity, effector capacity, and potential regulatory versus pathogenic roles, were not assessed and warrant further investigation.

Finally, although asymptomatic individuals are generally considered to progress less frequently to symptomatic malaria, the long-term protective versus pathological role of autoreactive antibodies in this context remains uncertain. Future longitudinal and mechanistic studies will be essential to determine whether these antibodies contribute to sustained parasite control, immune homeostasis, or potential immunopathology.

## Conclusions

In summary, this study demonstrates that asymptomatic *Plasmodium falciparum* infection in a high-transmission setting is associated with elevated autoreactive IgG responses, particularly of the cytophilic IgG3 subclass. The identification of conserved cytoskeletal and metabolic host proteins as dominant targets suggests that autoreactive antibody responses may represent a regulated feature of naturally acquired immunity rather than a nonspecific consequence of infection. The observed association between autoreactive IgG intensity and markers of mosquito exposure further highlights the role of environmental and transmission-related factors in shaping humoral immune repertoires in endemic areas. Together, these findings support the concept that autoreactive antibodies may serve as immunological correlates of cumulative exposure and clinical tolerance to malaria. While the functional relevance of these responses remains to be fully elucidated, our results provide a framework for future longitudinal and mechanistic studies aimed at defining the protective, regulatory, or pathological roles of autoreactive antibodies. Such work may ultimately contribute to the development of improved biomarkers for malaria exposure, immunity, and transmission intensity, thereby informing surveillance and control strategies in endemic regions.

## Supplementary Information

Supplementary Material 1.

## Data Availability

Data supporting the main conclusions of this study are included in the manuscript.
