# Supplementary material for: Autoreactive and parasite-specific IgG repertoires in asymptomatic and mild Plasmodium falciparum infection in Côte d’Ivoire
Source: Parasit Vectors. 2026 Jul 17;19:314. doi: 10.1186/s13071-026-07508-9 (PMC13435984; doi:10.1186/s13071-026-07508-9)
Supplement: Supplementary file 1 [file 13071_2026_7508_MOESM1_ESM.pdf]

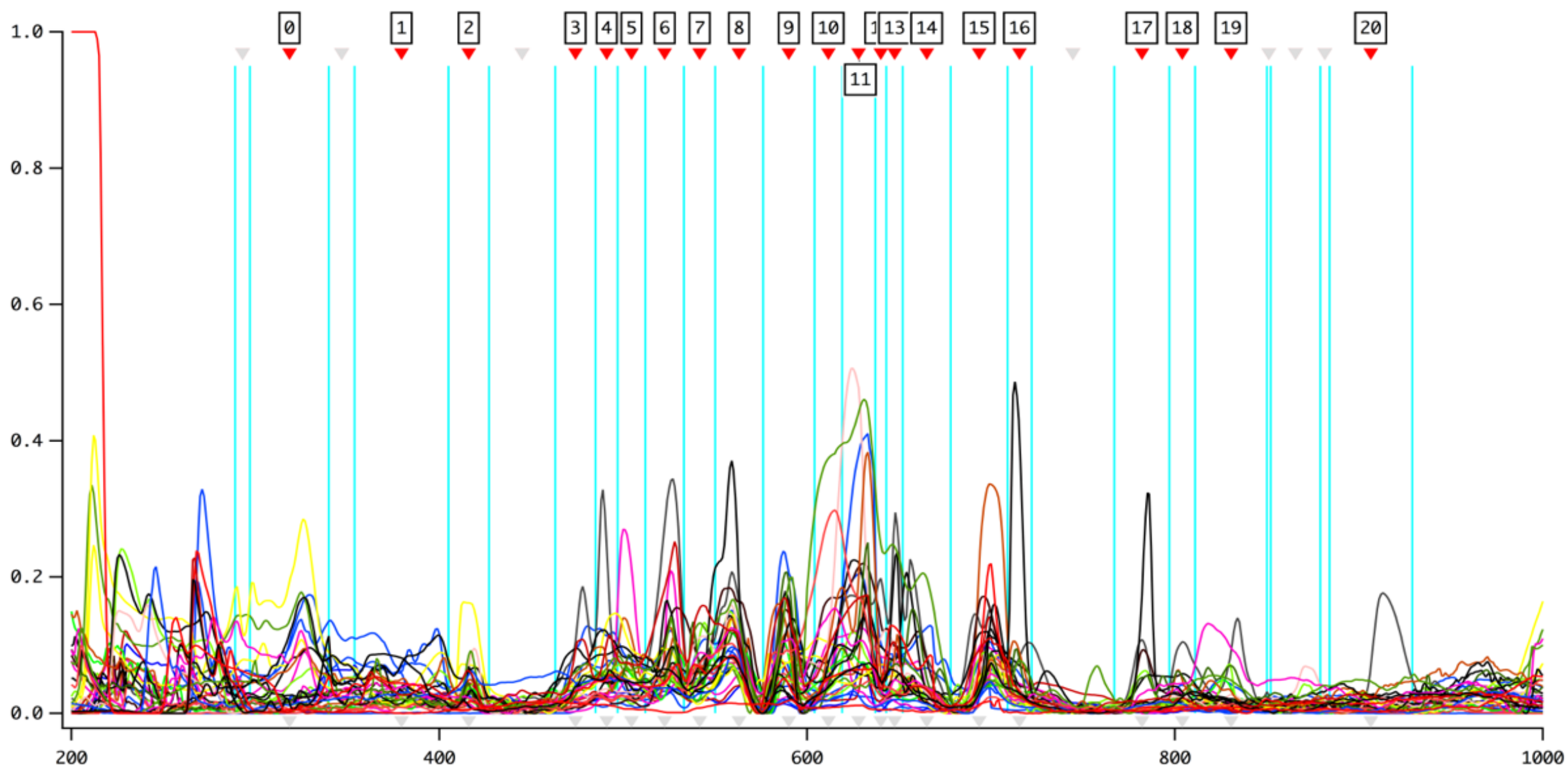

Supplementary Figure 1: Electrophoretic profile of self-reactive IgG of all participants from Abobo. Sections (band antigens) are defined in descending order from high molecular weight proteins to low molecular weight protein bands (250 to 20 kDa).

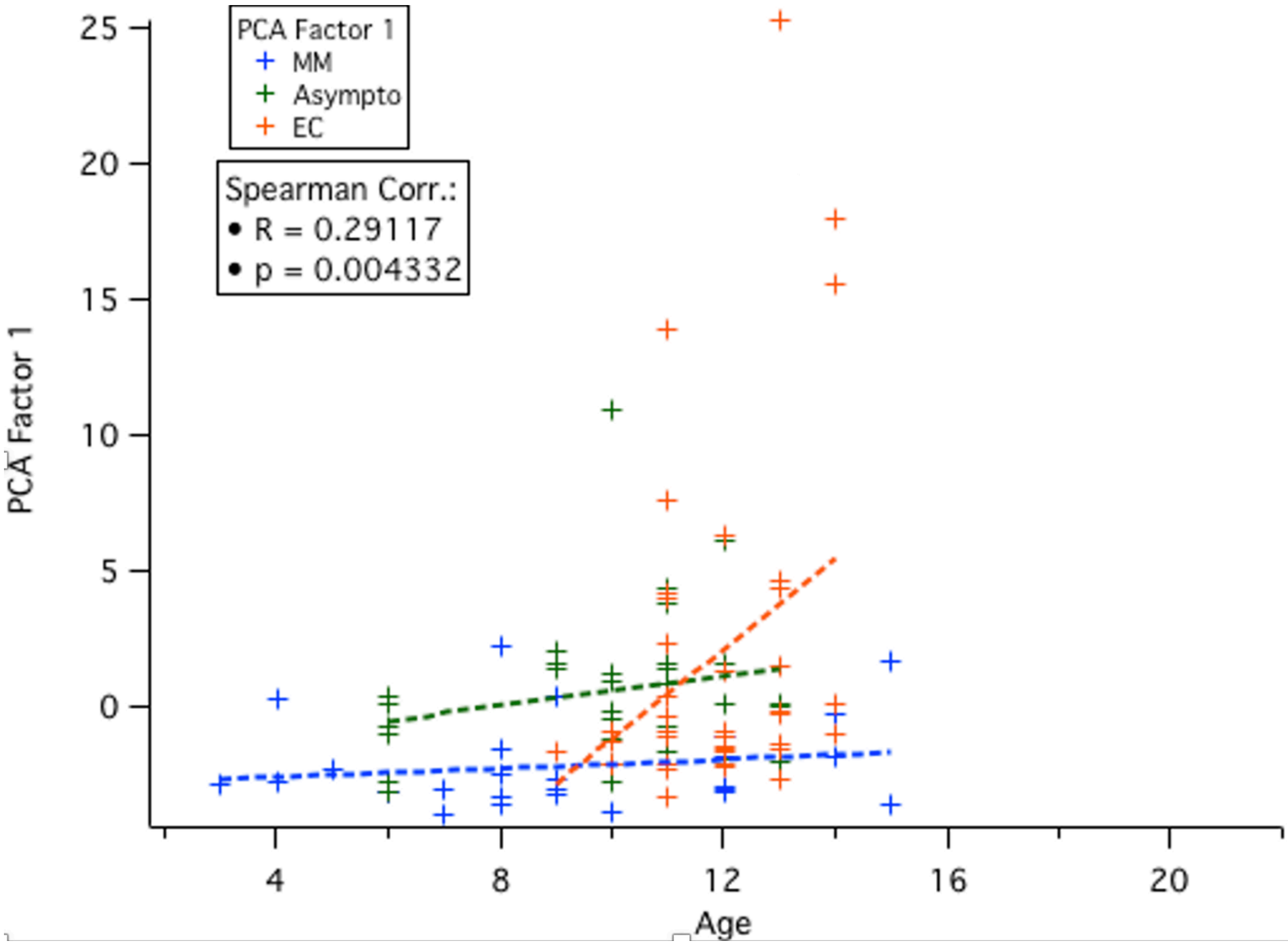

Supplementary Figure 2: Scatter plot showing the correlation between age and PCA-1 scores derived from brain self-reactive IgG reactivity patterns across patient subgroups.
